# Microarray analysis of embryo-derived bovine pluripotent cells: The vulnerable state of bovine embryonic stem cells

**DOI:** 10.1371/journal.pone.0173278

**Published:** 2017-03-03

**Authors:** Daehwan Kim, Yeon-Gil Jung, Sangho Roh

**Affiliations:** 1 Cellular Reprogramming and Embryo Biotechnology Laboratory, Dental Research Institute, Seoul National University School of Dentistry, Seoul, Republic of Korea; 2 ET Biotech Co. Ltd., Jangsu, Republic of Korea; University of Kansas Medical Center, UNITED STATES

## Abstract

Although there are many studies about pluripotent stem cells, little is known about pluripotent pathways and the difficulties of maintaining the pluripotency of bovine cells *in vitro*. Here, we investigated differently expressed genes (DEG) in bovine embryo-derived stem-like cells (eSLCs) from various origins to validate their distinct characteristics of pluripotency and differentiation. We identified core pluripotency markers and additional markers which were not determined as pluripotency markers yet in bovine eSLCs. Using the KEGG database, TGFβ, WNT, and LIF signaling were related to the maintenance of pluripotency. In contrast, some DEGs related to the LIF pathway were down-regulated, suggesting that reactivation of the pathway may be required for the establishment of true bovine embryonic stem cells (ESCs). Interestingly, oncogenes were co-down-regulated, while tumor suppressor genes were co-up-regulated in eSLCs, implying that this pattern may induce abnormal teratomas. These data analyses of signaling pathways provide essential information on authentic ESCs in addition to providing evidence for pluripotency in bovine eSLCs.

## Introduction

Pluripotent stem cells (PSCs) have two remarkable abilities: self-renewal and differentiation into various types of cells. Because of these unique characteristics, PSCs may be useful for replacement of diseased cells and tissues. Embryonic stem cells (ESCs) are a typical PSC and were first established in the mouse [1]. Since then, many studies have been conducted to generate ESCs in various species, including humans [2].

There also have been attempts to generate ESCs in domestic animals [3–6]. In agricultural perspective, the establishment of ESCs in domestic ungulates is able to provide a more efficient way to produce genetically-modified animals. Moreover, the ESCs are a valuable resource in many fields, such as biotechnology and biomedicine which may be represent an advantageous experimental tool for studying incurable or inherited diseases and developing therapeutic applications.

Cows are one of the most common and important domestic ungulates and are acknowledged as livestock for food and bioreactors [7]. There have been many attempts to establish ESCs in bovine species using general culture conditions for mouse ESCs (mESCs) and human ESCs (hESCs) [8–10]. However, those conventional methods are inappropriate for the survival of bovine ESCs (bESCs) *in vitro*, as they lose their stem cell properties when involved in proliferation, pluripotency, or differentiation. These early efforts to generate bESCs were gradually discontinued due to less than promising results which showed that the majority of the cultured cell population lacked evidence of pluripotency or ability to sustain long-term growth.

It was recently reported that mESCs were retained using leukemia inhibitory factor (LIF) along with the 2 inhibitor (2i) cocktail of PD0325901, a mitogen-activated protein kinase (MEK) inhibitor, and CHIR99021, a glycogen synthase kinase 3 (GSK3) inhibitor [11]. Moreover, in rats, which fail to maintain their pluripotency under conventional conditions [12], ESCs can be generated using 2i, as in mESCs [13]. There have been several attempts to generate authentic bESCs using small molecules. Recently, embryo-derived stem-like cells (eSLCs) from bovine embryos were successfully generated using three inhibitors (3i): PD18435 (MEK inhibitor), CHIR99021, and SU5402, a fibroblast growth factor receptor (FGFR) inhibitor [14, 15]. The eSLCs in 3i were able to proliferate and be maintained in culture for over 50 passages with the normal karyotype. In contrast to previous studies [9, 10], these cells expressed naïve pluripotency markers such as *REX1*, *KLF2*, *NROB1*, and *FGF4*. However, the cells were still defined as putative ESCs because of their incomplete capacity for *in vivo* differentiation, as they formed embryonic carcinomas instead of teratomas. Moreover, *CDX2*, the trophoblast-specific gene, is still expressed in the eSLCs. Recently, it has been reported that *CDX2*-knockdown embryo-derived stem cells are generated and have similar characteristics to genuine PSCs [16] although only one cell line was successfully established from 59 embryos. So far, many studies have failed to isolate true ESCs. However, there is limited information about the key aspects that fail during the establishment of bESCs, and little is known about their transcriptomes and biological functions. Although the eSLCs in this experiment are not complete bESCs, analyses of these cells can contribute to our understanding of the characteristics of embryo-derived PSCs in cattle.

Microarray technology has been used to analyze the differential gene expression of thousands of transcriptomes using short oligonucleotide probes, and the results provide unique global gene expression patterns. In stem cell research, microarray technology is used to confirm distinct characteristics of stem cells and analyze their functional performance using biological process (BP), molecular function (MF), and cellular component (CC) analysis [17]. To date, many genome-wide gene expression analyses of ESCs in humans and mice have been reported [18, 19]. The early results of those microarrays were analyzed to verify the differences among various embryo resources, including *in vitro* production (IVP), parthenogenesis (PA), and nuclear transfer (NT) [20, 21]. This technique has also been applied to compare somatic cells (SCs) with diverse stem cells from IVP, PA, and NT [22, 23]. Although two reports presented microarray data in cattle at the pre-implantation embryo level [24, 25], there are no reports of microarray data using bESCs.

In this study, we investigated the global gene expression patterns of bovine eSLCs from three different origins, IVP-, NT- and PA-embryos, to validate their distinct characteristics including pluripotency, imprinting, and chromatin remodeling. The study also demonstrated shared signaling pathways related to pluripotency. In addition, oncogenes and tumor suppressor genes were analyzed to understand the failure of teratoma formation in bovine ESCs.

## Materials and methods

### Chemicals

Most inorganic and organic compounds were purchased from Sigma-Aldrich Korea (Yong-in, Korea) and all liquid medium and supplements were from Life Technologies (Grand Island, NY, USA) unless indicated in the text.

### Oocyte recovery and *In Vitro* Maturation (IVM)

Bovine ovaries were collected from the Korean native beef cattle, HanWoo, at a local slaughterhouse (Livestock products market, Naju, Korea) and transported to the laboratory within 2–3 h of collection in saline at 25–35°C. Cumulus-oocyte complexes (COCs) were recovered by aspiration of 3 to 8 mm follicles. COCs that were enclosed by more than three layers of compact cumulus cells and an evenly granulated ooplasm were selected and incubated in IVM medium under warmed and gas-equilibrated mineral oil for 20–22 h at 38.5°C under 5% CO_2_. The IVM medium for oocytes is composed of tissue culture medium 199 with Earle’s salts and L-glutamine (TCM199) supplemented with 10% fetal bovine serum (FBS; Thermo Fisher Scientific Korea, Seoul, Korea), 10 μg/ml FSH-P (Folltropin-V^TM^, Vetrepharm, Belleville, ON, Canada), 0.2 mM sodium pyruvate, 1 μg/ml estradiol-17β, and 10 ng/ml epidermal growth factor.

### IVP of bovine fertilized embryos

IVP of bovine fertilized embryos was conducted as previously described [15]. The thawed HanWoo semen (purchased from HanWoo improvement center, Seosan, Korea) was deposited on the top of a discontinuous Percoll gradient prepared by depositing 2 ml of 90% Percoll under 2 ml of 45% Percoll in a 15 ml centrifuge tube, and the sample was then centrifuged for 20 min at 252 x *g*. The pellet was removed and re-suspended in 300 μl of hTALP and centrifuged at 201 x *g* for 10 min. The active semen from the pellet was inseminated with a matured oocyte for 24h (1 x 10^6^ sperm cells/ml). After insemination, the cumulus cells were removed by repeated aspiration into a pipette and denuded fertilized oocytes were transferred to *in vitro* culture medium consisting of CR2 with 0.3% ff-BSA and 1% ITS for 3 days. Oocytes were then transferred to CR2 medium with 0.15% ff-BSA, 1% ITS, and 0.15% FBS for 5 days at 38.5°C in a humidified gas environment of 5% CO_2_, 5% O_2,_ and 90% N_2_.

### Parthenogenesis and *in vitro* culture

Parthenogenetic activation was performed after IVM of the oocytes. The oocytes were activated in 5 μM Ca-ionophore for 5 min, followed by 2 mM 6-dimethylaminopurine (6-DMAP) for 3 h. After treatment, the activated oocytes were transferred and cultured *in vitro* as described above.

### Somatic cell nuclear transfer

The process of generating NT-embryos was conducted as previously described [14]. Briefly, matured oocytes were enucleated in HEPES-buffered TCM199 (hTCM199) supplemented with 20% FBS. The zona pellucida (ZP) was partially dissected with a fine glass needle to create a slit near the first polar body. The first polar body and the adjacent cytoplasm, presumably containing the metaphase II chromosomes, were extruded by squeezing with the needle. The enucleated oocytes were placed and incubated in hTCM199 with 10% FBS before NT.

A single donor cell isolated from ear skin tissue of the Korean native cattle, HanWoo, was injected into the perivitelline space of the enucleated oocyte through the slit made during enucleation. Then, karyoplast-cytoplast complexes were transferred into a cell fusion chamber with Zimmerman’s cell fusion medium and sandwiched between fine electrical rods. Cell fusion was accomplished with a single DC pulse of 25 V/mm for 10 μs. After 30 min of electric stimulation, fusion was confirmed under a stereomicroscope. The fused couplets were activated in 5 μM Ca-ionophore for 5 min, followed by 2 mM 6-DMAP for 3 h. After treatment, the activated oocytes were transferred and cultured *in vitro* as described above.

### Generation of embryo-derived Stem-Like Cells (eSLCs)

eSLCs were generated from three different origins (IVP-, NT- and PA-embryo) as previously described [14]. Briefly, ZP-free blastocysts were placed onto a mitomycin-C inactivated murine STO feeder cell layer and cultured at 38.5°C in a humidified gas atmosphere of 5% CO_2_ in 3i medium, which consists of equal volumes of DMEM/F12-Glutamax^TM^ and neurobasal media with 1% (v/v) N2 and 2% (v/v) B27 supplements plus the three inhibitors (3i): 0.8 μM PD184352 (Selleck Chemicals, Breda, Netherlands), 2 μM SU5402 (Tocris Bioscience, Ellisville, MO, USA), and 3 μM CHIR99021 (Tocris Bioscience). The colonies were passaged mechanically every 4 to 5 days and the medium was replaced every other day. Each colony from IVP-, NT- and PA-embryos was labeled I_x_-P_y_, N_x_-P_y_, and P_x_-P_y_ respectively along with its specific number x, P_y_ the passage number.

### Microarray gene expression analysis

For microarrays, the synthesis of target cRNA probes and hybridization were performed using Agilent’s Low RNA Input Linear Amplification kit (Agilent Technologies, Palo Alto, CA, USA) according to the manufacturer’s instructions. The fragmented cRNA was resuspended with 2X hybridization buffer and directly pipetted onto the assembled Agilent’s Bovine Oligo Microarray (44K). The arrays were hybridized at 65°C for 17 h using the Agilent Hybridization oven and the hybridized microarrays were washed as described in the manufacturer’s washing protocol (Agilent Technologies).

The hybridized images were scanned using Agilent’s DNA microarray scanner and quantified with Feature Extraction Software (Agilent Technologies). All data normalization and selection of fold-changed genes were performed using GeneSpringGX 7.3 (Agilent Technologies). The averages of normalized ratios were calculated by dividing the average of the normalized signal channel intensity by the average of the normalized control channel intensity. Hierarchical clustering was performed with TIGR MeV Ver.4.9 software (Institute of Genomic Research, Rockville, MD, USA) [26]. Microarray data are available from Gene Expression Omnibus (GEO, http://www.ncbi.nlm.nih.gov/geo/) with the accession number GSE92672.

### GO (Gene Ontology) annotation analysis

The functional annotation analysis of the co-up and downregulated gene lists was carried out using the Database for Annotation, Visualization and Integrated Discovery (DAVID, http://www.david.abcc.ncifcrf.gov/) based on GO annotation [27], as well as GO terms of BP, MF and CC. The annotation with a false discovery rate (FDR) was adjusted. *P* <0.05 was considered significant.

### Real-time (Quantitative) PCR

Total RNA from eSLCs, SCs, and ICM was extracted using the RNeasy mini kit (Qiagen, Valencia, CA, USA), and M-MLV Reverse Transcriptase was used to synthesize cDNA according to the manufacturer’s instructions. Real-time PCR was performed with a 7500HT system^TM^ (Applied Biosystems, Foster City, CA, USA) using SYBR Premix Ex Taq (Takara, Otsu, Japan). The PCR volume was 20 μl, containing 1 μl of reverse transcript product. Cycling conditions were 1 cycle of 95°C for 30 s, 40 cycles of 95°C for 5 s, and 60°C for 30 s. The specific primer sequences are listed in S1 Table.

### Statistical analysis

All values are expressed as mean ± SD. To determine the significance between two groups, comparisons were made using the Student’s *t*-test. Analysis of multiple groups was performed by one-way ANOVA using Graphpad Prism V5.0 (Graphpad Software. San Diego, CA, USA). *P* < 0.05 was considered significant.

## Results

### Comparison of embryo-derived Stem-Like Cells (eSLCs) and Somatic Cells (SCs)

**Fig 1:**
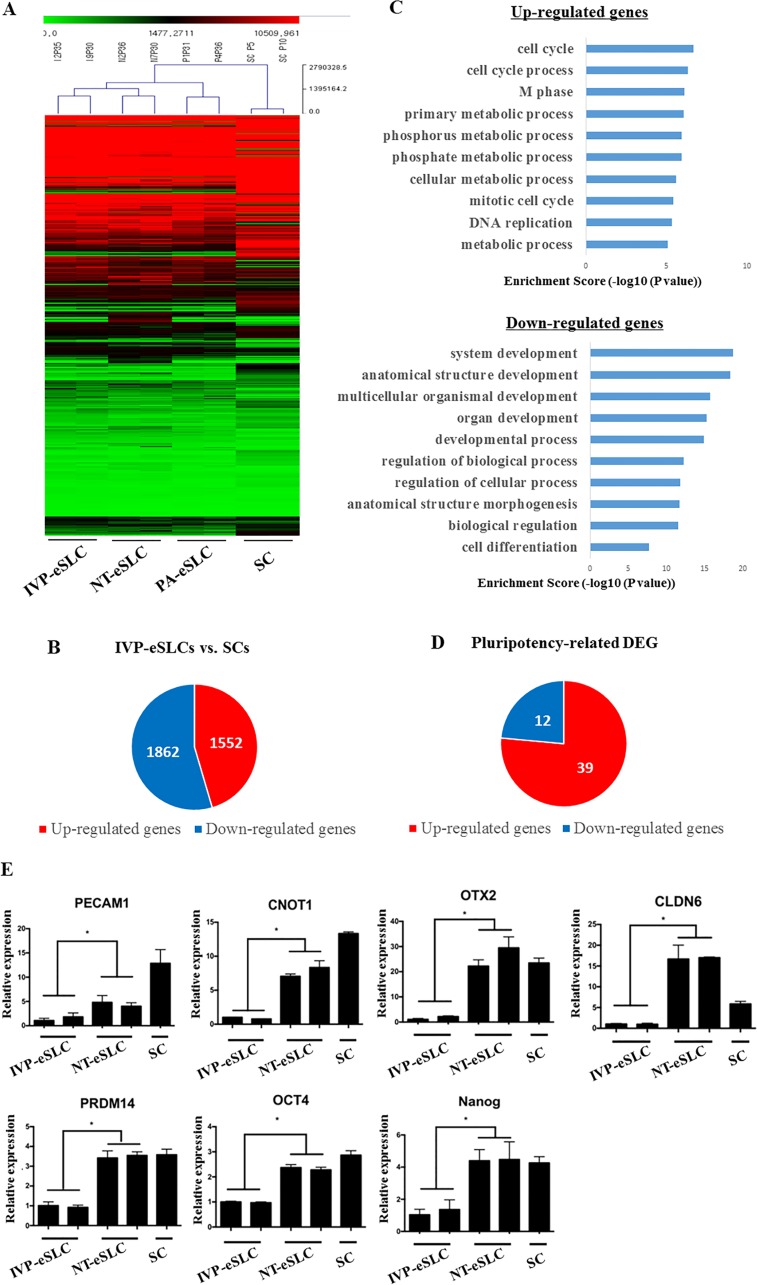
Comparison of gene expression between embryo-derived Stem-Like Cells (eSLCs) and Somatic Cells (SCs) in cattle. (A) Hierarchical cluster of *in vitro* production (IVP)-, nuclear transfer (NT)-, parthenogenesis(PA)-eSLCs, and SCs. The gene expression pattern from three eSLCs are countlessly different from SCs. (B) Venn diagram of all differently expressed genes (DEGs) in IVP-eSLCs and SCs. (C) Top 10 biological processes associated with significantly up-regulated and down-regulated genes in IVP-eSLCs and SCs. (D) Venn diagram of DEGs related to pluripotency in IVP-eSLCs and SCs. (E) Gene expression profiles of representative genes related to pluripotency. These genes are highly expressed in three eSLCs, compared with the genes in SCs. ICM is also presented as a control. *P<0.05 (n = 3).

To analyze the microarray data, we selected six different bovine eSLCs from three derivations of blastocysts: two IVP blastocysts, two NT blastocysts, and two PA blastocysts. The lists of differentially expressed genes (DEGs), determined using an absent/present (A/P) classification and ≥ 2-fold difference as cut-offs, are presented in S2 Table, and 10,203 genes were selected (Fig 1A).

These 10,203 genes were used to compare groups. To improve the accuracy of gene expression alteration as DEGs, we compared the normalized single value of each sample and the average value of each sample. Finally, significant differences in gene expression were confirmed by real-time PCR.

To investigate characteristics of bovine eSLCs, they were compared with SCs. Hierarchical clustering with the 10,203 genes showed that there was little difference in gene expression among the six different eSLCs. Conversely, all eSLCs had significantly different gene expression from SCs (Fig 1A).

### Differences between embryo-derived Stem-Like Cells (eSLCs) and Somatic Cells (SCs)

To further investigate specific differences between eSLCs and SCs, eSLCs from IVP-blastocysts (IVP-eSLCs) were selected as typical eSLCs, because they originated from an IVP-blastocyst produced by a sperm and an oocyte, similar to normal fertilization *in vivo*.

When we compared IVP-eSLCs and SCs, 3,414 genes were observed as DEGs: 1,552 of those genes were up-regulated and 1,862 genes were down-regulated (Fig 1B). There were 289 GO terms in the BP group that were enriched by adjusting the FDR (*P*<0.05) for up-regulated genes. The 10 dominant GO terms were listed and the most of them (9 of 10 terms) were related to metabolic activity or cell cycle (Fig 1C). There were also 419 GO terms in the BP group that were enriched by adjusting the FDR (*P*<0.05) for down-regulated genes. The 10 dominant GO terms were listed and the many of them (6 of 10 terms) were related to development or cell differentiation (Fig 1C). The top 10 most significantly up- or down-regulated DEGs in MF and CC are listed in S1 Fig.

To further investigate the properties of cultured IVP-eSLCs, we also analyzed pluripotency related genes. During the analysis, the microarray data were screened by GO terms (GO:0019827) related to stem cell maintenance.

Among the 144 genes, 39 genes were up-regulated and 12 genes were down-regulated (Fig 1D). Interestingly, these included core pluripotency markers including *OCT4* and *NANOG* as well as other markers that have not yet been identified in pluripotency, such as *PECAM1*, *CNOT1*, *CLDN6*, *FOXO1*, *PRDM14*, and *OTX2* (Fig 1D). The fold changes of the genes were also presented in S2 Fig. These genes were also confirmed by real-time PCR (Fig 1E).

### Gene expression profiles among embryo-derived Stem-Like Cells (eSLCs) from three different origins

**Fig 2:**
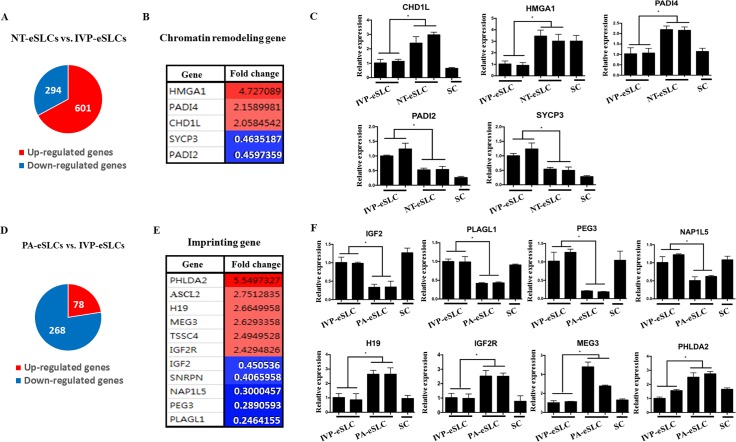
Comparison of Differently Expressed Genes (DEGs) among embryo-derived Stem-Like Cells (eSLCs) from three different origins. (A) Venn diagram of all DEGs in nuclear transfer-eSLCs (NT-eSLCs) and *in vitro* production-eSLCs (IVP-eSLCs). (B) Chromatin remodeling genes in NT-eSLCs and IVP-eSLCs. (C) Gene expression profiles of DEGs related to chromatin remodeling. (D) Venn diagram of all DEGs in parthenogenesis-eSLCs (PA-eSLCs) and IVP-eSLCs. (E) Imprinting genes in PA-eSLCs and IVP-eSLCs. The expression of paternally expressed imprinting genes is increased in PA-eSLCs compared with the gens in IVP-eSLCs, while maternally expressed imprinting genes are *vice versa*. (F) Gene expression profiles of DEGs related to imprinting. Somatic cells are also presented as a control. *P<0.05 (n = 3).

In order to further investigate characteristics of eSLCs, IVP-eSLCs were compared with PA- or NT-eSLCs. First, we examined the pattern of DEGs between NT- and IVP-eSLCs and identified 895 DEGs, with 601 up-regulated and 294 down-regulated genes (Fig 2A). The top 10 most significantly up- or down-regulated DEGs in the BP, MF, and CC are also listed in S3 Fig. Although 77 chromatin remodeling related genes (GO:0006338) were not in the major group, they were also profiled between NT- and IVP-eSLCs. Only 5 genes, *HMGA1*, *PADI4*, *CHD1L*, *SYCP3*, and *PADI2*, were revealed as DEGs in this study (Fig 2B), and their expression patterns were confirmed by real-time PCR (Fig 2C).

Next, the gene expression pattern in between PA- and IVP-eSLCs was analyzed. A total of 346 genes were differently expressed between PA- and IVP-eSLCs, with 78 up-regulated genes and 268 down-regulated genes (Fig 2D). The top 10 most significantly up- or down-regulated DEGs in the BP, MF, and CC are also listed in S4 Fig. Although these were not in the major group, 12 imprinting related genes were included in these DEGs (Fig 2E). Surprisingly, among these genes, PA-eSLCs had higher expression of *PHLDA2*, *ASCL2*, *H19*, *MEG3*, *TSSC4*, and *IGF2R* as imprinted maternally expressed genes than IVP-eSLCs (Fig 2F). On the other hand, the expression of 5 imprinted paternally expressed genes, *IGF2*, *SNRPN*, *NAP1L5*, *PEG3*, and *PLAGL1*, was down-regulated in PA-eSLCs compared to IVP-eSLCs (Fig 2E). These genes were also confirmed by real-time PCR (Fig 2F).

### The expectation of signaling pathways for bovine pluripotency

**Fig 3:**
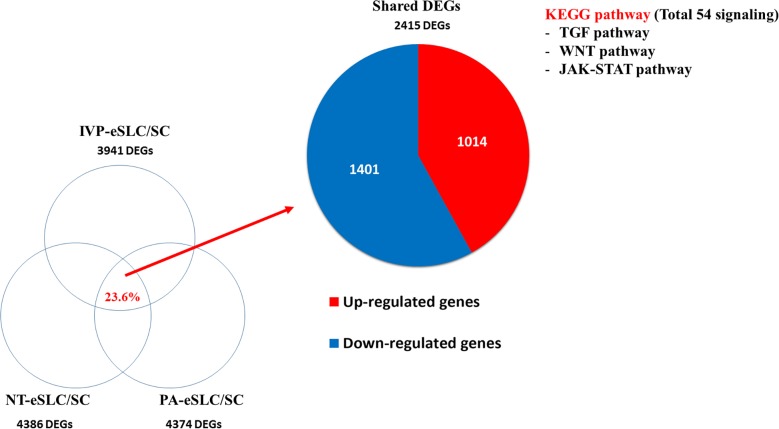
Differently Expressed Genes (DEGs) in embryo-derived Stem-Like Cells (eSLCs) and the analysis of distinct pathways related to pluripotency. In total of 10203 genes, the DEG numbers of *in vitro* production (IVP)-, nuclear transfer (NT)-, parthenogenesis (PA)-eSLCs are 3941, 4386 and 4374, respectively. Among them, co-expressed DEGs are 2415 (23.6%). By KEGG analysis of the co-expressed DEGs, there are 54 signaling including TGF-β, WNT, and LIF pathways which are strongly related to pluripotency.

**Fig 4:**
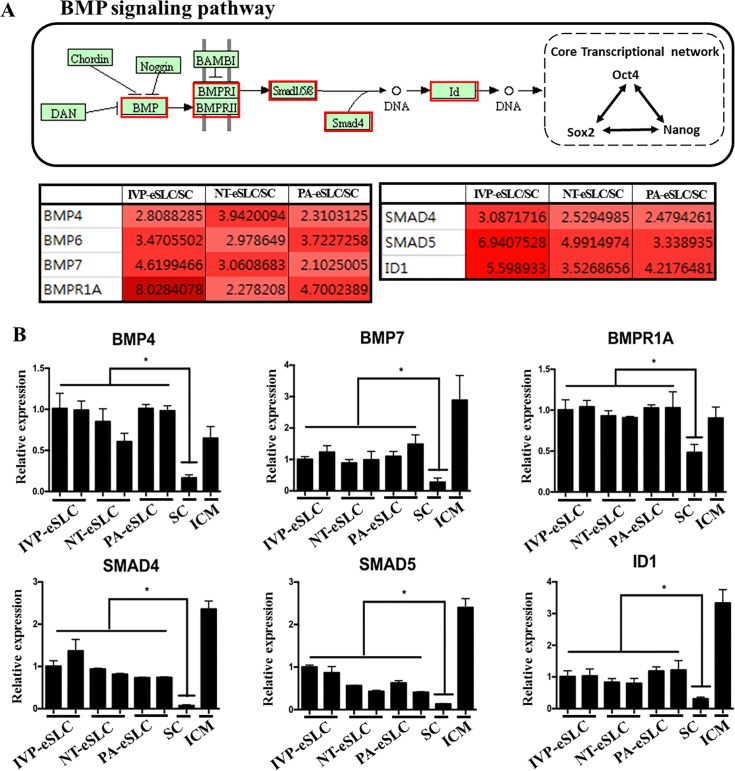
BMP signaling pathway in embryo-derived Stem-Like Cells (eSLCs). (A) KEGG pathway map of BMP signaling related to core transcriptional network for pluripotency. Most differently expressed genes (DEGs) related to BMP signaling are up-regulated in eSLCs compared with the gens in somatic cells. The boxes outlined with red indicate relatively up-regulated DEGs. Fold change value is also provided with red in the table below (A). (B) Gene expression profiles of DEGs related to the BMP signaling pathway. ICM and somatic cell (SC) are also presented as a control. *P<0.05 (n = 3).

**Fig 5:**
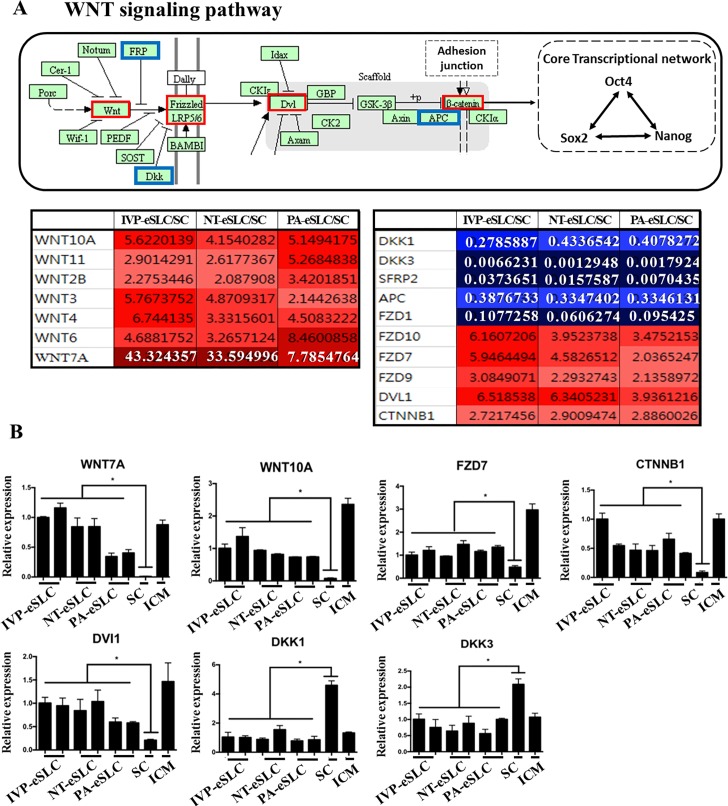
The WNT signaling pathway in embryo-derived Stem-Like Cells (eSLCs). (A) KEGG pathway map of WNT signaling related to core transcriptional network for pluripotency. Most differently expressed genes (DEGs) related to WNT signaling in eSLCs are up-regulated when compared with somatic cells (SCs), although some genes, such as *FZD1* and *APC*, are down-regulated. *DKK1*, *DKK3*, and *SFRP2*, inhibitors of BMP signaling, are down-regulated in eSLCs. The boxes outlined with red indicate relatively up-regulated DEGs, while the ones outlined with blue point to relatively down-regulated DEGs. Fold change value is also provided with red (up-regulated genes) and blue (down-regulated genes) in the tables below (A). (B) Gene expression profiles of representative DEGs related to the BMP signaling pathway. ICM and somatic cell (SC) are also presented as a control. *P<0.05 (n = 3).

**Fig 6:**
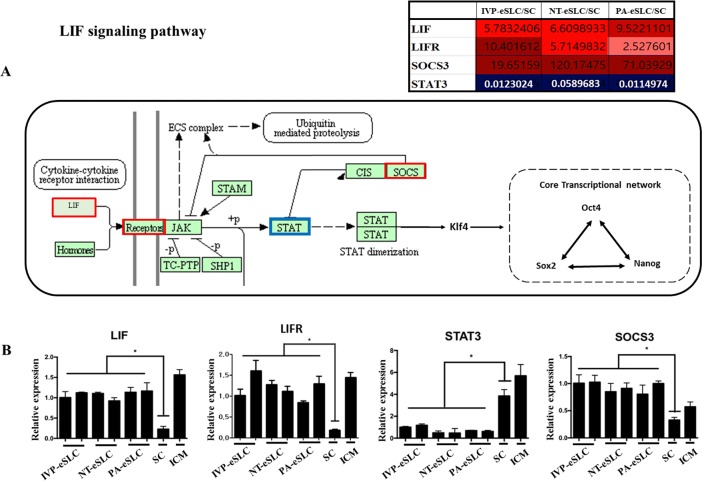
LIF signaling pathway in embryo-derived Stem-Like Cells (eSLCs). (A) KEGG pathway map of LIF signaling related to core transcriptional network for pluripotency. *LIF*, *LIFR*, and *SOCS3* genes are up-regulated in eSLCs when compared with somatic cells, while the *STAT3* gene is down-regulated. The boxes outlined with red indicate relatively up-regulated DEGs, while the ones outlined with blue mark relatively down-regulated DEGs. Fold change is also provided with red (up-regulated genes) and blue (down-regulated genes) in the table above (A). (B) Gene expression profiles of DEGs related to the LIF pathway. ICM and somatic cell (SC) are also presented as a control. *P<0.05 (n = 3).

Although there are many studies of stem cells, little is known about the signaling pathways related to pluripotency in bovines. Therefore, the co-expression pattern of whole genes in eSLCs may be a valuable tool for the discovery of important pathways related to pluripotency in bovines. To elucidate these pathways in more detail, we specifically searched for co-expressed genes that may be related to signaling pathways for pluripotency, and the biological pathways were analyzed by the Kyoto Encyclopedia of Genes and Genomes (KEGG) database in bovines [28]. In co-up-regulated genes among eSLCs, we identified 2,415 DEGs, with 1,014 co-up-regulated genes and 1,401 co-down-regulated genes (Fig 3). By the KEGG database, there were 54 signaling pathways in DEGs, and some of them were related to the maintenance of pluripotency, including TGFβ, WNT, and LIF signaling (Fig 3). In TGFβ signaling, the BMP family and SMAD family were contained in DEGs and several key genes were confirmed by real-time PCR (Fig 4). In WNT signaling, 19 genes such as *WNT7a*, *WNT10a*, *FZD7*, *DKK1*, and *DVI1* were included in DEGs, and core genes were confirmed by real-time PCR (Fig 5). In LIF signaling, *LIF*, *STAT3*, and *SOCS3* were identified as DEGs and confirmed by real-time PCR (Fig 6).

### The expression pattern of tumor-related genes in bovine embryo-derived Stem-Like Cells (eSLCs)

**Fig 7:**
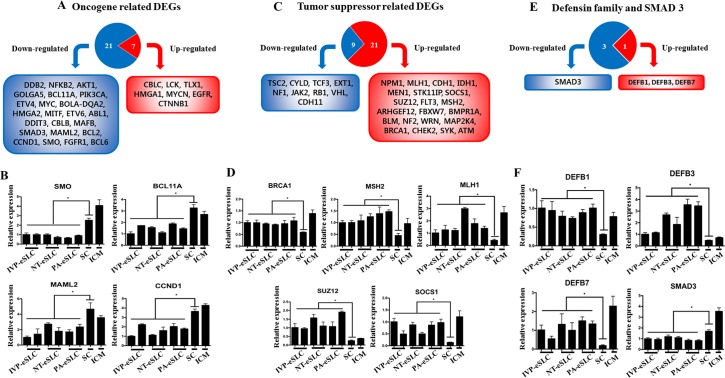
Oncogene- and tumor suppressor-related Differently Expressed Genes (DEGs) in embryo-derived Stem-Like Cells (eSLCs). (A) Venn diagram shows 7 up-regulated and 23 down-regulated DEGs related to oncogenes. (B) Gene expression profiles of representative differently expressed oncogenes. (C) Venn diagram shows 21 up-regulated and 9 down-regulated DEGs related to tumor suppressors. (D) Gene expression profiles of representative differently expressed tumor suppressors. (E) Venn diagram shows DEGs related to the Defensin family (tumor suppressor) and *SMAD3* (oncogene). (F) Gene expression profiles of DEGs related to the Defensin family and *SMAD3*. ICM and somatic cells (SCs) are also presented as a control. *P<0.05 (n = 3).

To elucidate abnormal teratoma formation in bovine eSLCs, we attempted to examine 82 oncogenes and 63 tumor suppressor genes among eSLCs, as described on the Cancer Genes website and via literature searches [29]. Among the oncogenes, 7 co-up-regulated genes and 23 down-regulated DEGs were identified in eSLCs (Fig 7A). The fold changes of the genes were also presented in S5 Fig. The expression of key genes in down-regulated DEGs was confirmed by real-time PCR (Fig 7B). Among tumor suppressors, 30 DEGs, with 21 co-up-regulated genes and 9 co-down-regulated genes were also identified (Fig 7C), and the fold changes of the genes were provided in S6 Fig. The expression of primary genes in up-regulated DEGs was confirmed by real-time PCR (Fig 7D).

We also investigated *DEFB1*, *DEFB3*, *DEFB7*, and *SMAD3*, which may be related to teratoma formation. Interestingly, according to our data, *SMAD3* expression was decreased, while the expression of *DEFB1*, *DEFB3*, and *DEFB7* was increased in eSLCs compared to SCs (Fig 7E). The fold changes were also listed in S7 Fig. These genes were also confirmed by real-time PCR (Fig 7F).

## Discussion

The microarray technology has revealed a powerful tool for profiling the global gene expression and DEGs are suggested specific or universal characteristics. To understand their characteristics, PSCs in many species including humans and mice have been analyzed by this technology. However, it is still not well known yet about gene expression profiles of embryo-derived PSCs in cattle. In the present study, we analyzed the gene expression pattern of bovine eSLCs from three different origins: IVP-, NT-, and PA-blastocysts. These were compared with each other to understand their shared and distinct properties. In addition, these were compared with SCs to understand shared pathways for pluripotency and failure of teratoma formation by profiling tumor-related genes and tumor suppressor genes.

To understand characteristics of eSLCs and SCs, we analyzed their gene expression. The hierarchical clustering results show little difference in gene expression among six different eSLCs, while all the eSLCs have immensely different gene expression from SCs (Fig 1A), suggesting that properties of eSLCs may be significantly different from those of SCs.

To further verify differences between eSLCs and SCs in detail, IVP-eSLCs were compared with SCs. Among up-regulated DEGs, most GO terms in the BP group were related to metabolic activity and the cell cycle (Fig 1C). Generally, the cell cycle of ESCs is shorter than that of SCs, because the durations of G1 and G2 are remarkably decreased [30]. This means that there is a rapid onset of stem cell proliferation and an enormous demand for energy, such as ATP. Because of this, the metabolic system in ESCs is also changed [31]. Consequentially, metabolism-related genes in ESCs were up-regulated compared to SCs. The results suggest that the metabolic system of IVP-eSLCs may be similar to ESCs and that they may have a short cell cycle, consistent with our previous report [14].

Expression of pluripotent genes and inhibition of differentiation genes are both necessary for maintenance of a pluripotent state. Recently, it has been documented that mESCs are able to maintain their unique properties, including self-renewal and potential of differentiation, by using inhibitors which suppress differentiation signaling pathways [11]. More recently, bovine eSLCs were also derived with these inhibitors [14]. According to our results in down-regulated DEGs, most GO terms in the BP group were related to differentiation and development (Fig 1C). These results suggest that the expression of differentiation-associated genes in bovine eSLCs is decreased when compared with SCs, suggesting that the 3i system may repress the tendency to differentiate in bovine eSLCs. These results may help to retain pluripotency in eSLCs.

One of the biggest differences between ESCs and SCs is the expression of pluripotent genes [32]. Comparing with SCs, eSLCs expressed 39 pluripotent DEGs including the core pluripotency markers, *OCT4* and *NANOG*. It has been documented that *OCT4* and *NANOG* expressions are essential not only to decide first cell fate, trophoblast and inner cell mass, but also to maintain pluripotency of stem cells in mouse, as well as human [33, 34]. In bovine, it has been also revealed that *OCT4* and *NANOG* are also expressed in embryos and embryo-derived cells [14]. Moreover, the overexpression of two genes was essential to generate bovine induced pluripotent stem cells [35]. These previous reports and our results suggest that *OCT4* and *NANOG* expressions may be indispensable to support bovine ESCs. Among these DEGs, some pluripotency related genes are well known in mESCs and hESCs, but have not yet been reported in bovine embryo-derived cells; these include *PECAM1*, *CNOT1*, *OTX2*, *PRDM14*, and *CLDN6* (Fig 1D). These genes have been well-known as pluripotency markers in mESCs [36–40]. In this study, these up-regulated DEGs were also confirmed by real-time PCR and the results revealed that these genes were significantly increased in IVP-eSLCs compared to SCs (Fig 1E). Surprisingly, their expression was similar in IVP-eSLCs and ICM, implying that these genes may act as pluripotency markers and can aid in distinguishing the population of true pluripotent stem cells in bovines.

Recent evidence suggests that ESCs from NT- and PA-embryos contribute epigenetic modifications such as chromatin remodeling and imprinting [41, 42]. This suggests that the analysis of bovine eSLCs from NT- and PA-embryos might be useful for predicting epigenetic deficiencies that induce unsuccessful development.

Although NT-embryos are produced by oocyte-derived reprogramming factors like IVP-embryos, the efficiency was extremely low and transcriptional abnormalities were revealed [43]. The major cause of these developmental failures may be due to epigenetic modifications such as chromatin remodeling [44]. Profiling of chromatin remodeling-related genes revealed 5 genes that were differentially expressed between IVP- and NT-eSLCs (Fig 2B and 2C). The expression of *HMGA1*, *PADI4*, and *CHD1L* was significantly increased in NT-eSLCs compared to IVP-eSLCs (Fig 2B and 2C). Interestingly, these genes were not only related to chromatin remodeling, but were also associated with pluripotency. [45–47]. They may be sufficient to trigger a cascade of epigenetic problems, leading to low efficiency of differentiation and development of the NT-embryo, despite the small number of DEGs.

Comparisons between PA- and IVP-eSLCs revealed differences in imprinting gene expression, which is consistent with a previous study [23]. Although the expression of some genes did not increase exactly two fold, an increasing trend for imprinted maternally expressed genes and a decreasing trend for imprinted paternally expressed genes in PA-eSLCs by real-time PCR were observed when compared to IVP-eSLCs (Fig 3E and 3F). PA-eSLCs maintained an abnormal expression pattern of imprinting-related genes, like the PA embryo, and may be useful for preventing the waste of embryos in imprinting studies.

Since establishing mESCs, many studies have generated ESCs in bovines [48–50]. However, there have been no reports that identify signaling pathways that maintain pluripotent stem cells. In order to verify the appropriate pathways associated with bovine pluripotency, we investigated and analyzed co-regulated genes among eSLCs with the KEGG database. According to our results, co-regulated genes associated with pluripotency are strongly related to the TGFβ, WNT, and LIF signaling pathways (Fig 3).

Although BMP signaling, which belongs to the TGFβ superfamily, promotes non-neural differentiation, BMPs also maintain pluripotency by activation of inhibitor of differentiation (Id) genes [51]. In addition, in mice, BMPs are able to support pluripotency in the absence of both serum and feeder cells [52]. Moreover, recent evidence has revealed that BMP4 plays an indispensable role in establishing bovine iPSCs [53]. Although some genes were down-regulated in this study, core BMP signaling genes appeared in co-up-regulated DEGs. In particular, *BMP4*, *BMP7*, *BMPR1A*, *SMAD4*, *SMAD5*, and *Id1* were up-regulated (Fig 4A and 4B). Interestingly, the expression pattern of these genes in all eSLCs was similar to that of ICM, suggesting that BMP signaling may be activated and may support pluripotency of bovine eSLCs, similar to the role of ICM in embryos.

The WNT pathway is also important for the enhancement of proliferation and maintenance of pluripotency in ESCs, as it stabilizes cytoplasmic b-catenin by suppressing GSK3β [54]. According to our results, WNT signaling genes such as *WNT7a*, *WNT10a*, and *FZD7* were involved in co-up-regulated DEGs (Fig 5A and 5B). Moreover, *DVL1* and *β-CATENIN*, which are downstream of WNT signaling, are also expressed highly in bovine eSLCs. On the other hand, *DKK1* and *DKK3*, which are suppressors of WNT signaling, were down-regulated in bovine eSLCs (Fig 5A and 5B). Interestingly, this gene expression pattern was similar to that revealed in ICM (Fig 5B). These results suggest that the WNT pathway may be activated as one of the strongest regulators to support pluripotency in bovine eSLCs.

Recently, it has been documented that LIF signaling is essential in mESCs and naïve hESCs [55]. In our results, LIF signaling also appeared in DEGs. The expression of *LIF* and *LIFR* genes was up-regulated, while *STAT3* expression was down-regulated in eSLCs (Fig 6A). Surprisingly, the expression of STAT3 in eSLCs was in reverse of its expression in ICM, suggesting that the signal transmission between LIF and STAT3 may be disconnected. It has been reported that many culture systems in previous studies, even those including LIF, fail to generate true bESCs [8–10]. According to our results, the failure may be related with the disconnection between LIF and STAT3. For the maintenance of pluripotency in bESCs, the LIF signaling pathway may be activated by STAT3 signaling and/or downstream effectors which do not supplement or stimulate LIF itself.

SOCS3 inhibits JAK signaling by a binding mechanism, resulting in the inhibition of the LIF pathway for pluripotency [56]. According to our microarray and real-time PCR results, *SOCS3* expression was significantly up-regulated in eSLCs compared to SCs (Fig 6). Assuming that the increased expression of *SOCS3* may inhibit JAK signaling, this may be a critical factor involved in destruction of the LIF pathway. This study therefore suggests that the reactivation of *STAT3* may be compulsory for establishment of true ESCs in bovines, and *SOCS3* inhibition may generate authentic bESCs.

Generating eSLCs in a 3i culture system with long-term proliferation and expression of pluripotent markers has been previously successful. However, the efficiency of *in vivo* differentiation is extremely low and teratoma formation was induced abnormally [14]. Many previous studies in the literature have also revealed similar problems [48, 57, 58], even in bovine iPSCs [35].

It was hypothesized that tumor-related genes may be changed in eSLCs, so we profiled oncogenes and tumor suppressor genes. Interestingly, among DEGs, most oncogenes (23 genes) were down-regulated, including *SMO*, *BCL11a*, *MAML2*, and *CCND1* which are related to tumors and metastasis [59–62] (Fig 7A). These results suggest that decreased oncogenes may reduce the frequency of teratoma formation and immature teratomas. In contrast, most tumor suppressor genes (21 of 30 genes) were highly up-regulated in eSLCs including *BRCA1*, *MLH1*, *MSH2*, *SUZ12*, and *SOCS1*, which are related to an increased risk of cancer [63–67]. These results indicate that up-regulation of these tumor suppressor genes may be associated with suppression of teratoma formation in bovine eSLCs.

Some genes that affect teratoma formation are not tumor-related. The defensin family is a well-known immune system-connected factor [68] that can suppress tumor formation [69]. We observed increased expression of defensin family genes including *DEFB1*, *DEFB3*, and *DEFB7* (Fig 7E and 7F). These results show that defensin family genes may also be candidates for teratoma formation in bovines. It has also been documented that SMAD3 is the mediator of signals from the TGFβ superfamily, which controls cell proliferation, pluripotency, and differentiation [70]. Recently, it has been reported that SMAD3 is closely connected with teratoma formation from ESCs [71]. *SMAD3* expression was down-regulated (Fig 7E and 7F). It is speculated that decreased *SMAD3* gene expression may be one of the reasons why teratomas are induced abnormally.

In conclusion, our study demonstrate that expression of oncogenes were predominantly decreased, while tumor suppressor genes were increased in bovine eSLCs, compared with that in SCs. This indicates that the ability of bovine eSLCs in 3i and previous culture conditions to form teratomas may be eroded by the regulation of oncogene and tumor suppressor gene expression. In view of these findings, further investigation of oncogenes in bovine embryo-derived cells may be useful for the generation of genuine bovine ESCs.

## Conclusions

Our report illustrates gene expression patterns of three different eSLCs from IVP-, NT-, and PA-embryos. To the best of our knowledge, this study represents the first report of gene expression profile data obtained from the DNA microarray analysis in bovine embryo-derived PSCs. Data analyses of signaling pathways provide essential information on authentic ESCs as well as supporting evidence for pluripotency in bovine eSLCs. Moreover, the gene expression profiles of eSLCs from various types of blastocysts can also provide insight into common and/or specific behavior patterns of genomes and epigenomes, particularly in domestic mammalian species.

## Supporting information

S1 Fig Functional annotation analysis between *in vitro* production embryo-derived stem-like cells and somatic cells. The top 10 most significantly up-regulated and down-regulated differently expressed genes in molecular function and cellular component are shown with tables and corresponding bar graphs.
(XLSX)

S2 Fig Profiling of up-regulated and down-regulated differently expressed genes between *In Vitro* Production embryo-derived Stem-Like Cells (IVP-eSLCs) and Somatic Cells (SCs). Among stem cell maintenance related genes, 39 genes were up-regulated (Red) and 12 genes were down-regulated (blue).
(XLSX)

S3 Fig Comparison analysis of the functional annotation between Nuclear Transfer embryo-derived Stem-Like Cells (NT-eSLCs) and *In Vitro* Production embryo-derived Stem-Like Cells (IVP-eSLCs). The top 10 most significantly up-regulated and down-regulated differently expressed genes in biological process, molecular function and cellular component are shown with tables and corresponding bar graphs.
(XLSX)

S4 Fig Comparison analysis of the functional annotation between Parthenogenesis embryo-derived Stem-Like Cells (PA-eSLCs) and *In Vitro* Production embryo-derived Stem-Like Cells (IVP-eSLCs). The top 10 most significantly up-regulated and down-regulated differently expressed genes in biological process, molecular function and cellular component are shown with tables and corresponding bar graphs.
(XLSX)

S5 Fig Oncogene-related differently expressed genes in embryo-derived stem-like cells. Fold changes of 7 co-up-regulated (red) and 23 co-down-regulated (blue) genes are listed. All fold change was normalized by values of somatic cells.
(XLSX)

S6 Fig Tumor suppressor-related differently expressed genes in embryo-derived stem-like cells. Fold changes of 21 co-up-regulated (red) and 9 co-down-regulated (blue) genes are listed. All fold change was normalized by values of somatic cells.
(XLSX)

S7 Fig Gene profiling of defensin family and SMAD3 in embryo-derived stem-like cells. DEFB1, DEFB3 and DEFB7 were up-regulated as tumor suppressor genes (red) in eSLCs, while SMAD3 was down-regulated as oncogenes (blue). All fold change was normalized by values of somatic cells.
(XLSX)

S1 Table Primer sequences for real time polymerase chain reaction. (DOCX)

S2 Table Microarray data for bovine embryo-derived stem-like cells and somatic cells. (XLSX)
