# Supplementary material for: Microarray analysis of embryo-derived bovine pluripotent cells: The vulnerable state of bovine embryonic stem cells
Source: PLoS One. 2017 Mar 3;12(3):e0173278. doi: 10.1371/journal.pone.0173278 (PMC5336296; doi:10.1371/journal.pone.0173278)
Supplement: S1 Table — (DOCX) [file pone.0173278.s008.docx]

Table S1. Primer sequences for Real Time Polymerase Chain Reaction

| Group | Gene name | Sequence | | Reference |
| --- | --- | --- | --- | --- |
|  |  | Forward | Reverse |  |
| Pluripotency | NANOG | CAGGGACTATGGAGCTCAGG | CTGGATGCTGACAATCATGG | (Kim et al., 2015) |
|  | OTX2 | CAATGCAGTCACCAGCCATCTCAA | TGCCTCCTGGGACATTGATCA | (Smith et al., 2007) |
|  | PECAM1 | CTCACCAAGGTCTGGGAACAA | TCTTCTCGGAATGTGGGCAT | This study |
|  | CNOT1 | TTG GAC AGA TGC ACC AGC AA | AGT CGG ACA AAG GCA TCC AG | This study |
|  | PRDM14 | ATTTTCGTTCCGCCGCCCCC | TCAGCCCCGGCGCTATCGGT | (McLean et al., 2014) |
|  | CLDN6 | ACAAGCCTTTCCTTGCTGGTCA | AGTAACAACTGGTGAGTGTGGGCA | (Cagnone and Sirard, 2014) |
|  | OCT4 | GCCTGGATTTTCCTAGCATCTAC | GGCCAAGCAGGCTTTG | (Kim et al., 2015) |
| Imprinting | PHLDA2 | GCTCCAGGTGTGGAAGAAGA | GACGCGTTCCAGTAGCTCTC | (Jiang et al., 2015) |
|  | H19 | CTTGGAACACGGACTTCTTCAAG | TCTACTTCAGCCGACCATCCA | (Yang et al., 2005) |
|  | MEG3 | GTTTGGGACTGGGACGCTTA | GGAGACGACGGACAGAGTTC | This study |
|  | IGF2R | CCGGGAGATGGTAATGAGCA | TCTCGTTCTCGTCGGCCT | (Yang et al., 2005) |
|  | IGF2 | TCTACTTCAGCCGACCATCCA | GTAAGTCTCCAGCAGGGCCA | (Yang et al., 2005) |
|  | NAP1L5 | TCC AAC TGT GTG TCC CCA TC | ACG AAG GCA CAG CTA ACA CA | This study |
|  | PEG3 | CGCCAAAGTCAGGGAGAG | CTTAACTGCCAGGACACC | (Katz-Jaffe et al., 2009) |
|  | PLAGL1 | TCTCTAAGGTTCTTCCCTGCCT | TAAGCTTGGGGTCCAGAGGA | This study |
| Oncogene | SMO | GCTTCACCCGTCTACTACCC | GCTCATGGAAATGCCAGTTC | (Zhang et al., 2014) |
|  | BCL11A | GGG TAT TTG TAA AGA TGA GCC CAG | GGG GTG TGT GAA GAA CCC G | This study |
|  | CCND1 | AGCAGAAGTGCGAGGAAGAG | GCCAGGTTCCACTTGAGTTT | (Shimizu et al., 2013) |
|  | MAML2 | CCC AAC CCC TGC TCA AAT CC | GCA ATT TTC TCC GCA TCA GCC | This study |
| Tumor  suppressor | MLH1 | TCCGGGAGATGCTGCATAA | CAA GGC CCA CTG AGG ATT CA | (Mamo et al., 2011) |
|  | MSH2 | AGACAGGTCGGAGTTGGGTA | CCTCCTCTCTGAATAACCTGCC | This study |
|  | SOCS1 | CTCGTACCTCCTACCTCTTCATGTT | ACAGCAGAAAAATAAAGCCAGAGA | (Vitorino Carvalho et al., 2014) |
|  | SUZ12 | CATCCAAAAGGTGCTAGGATAGATG | TGGGCCTGCACACAAGAATG | (Rekawiecki et al., 2012) |
|  | BRCA1 | GGA GCC CTC ATC ATT CAC CC | CCC GAT CAC ATG GAA GCC AG | This study |
| Chromatin | HMGA1 | GCCTCCAAGCAGGAAAAGG | TTGCTTCCCTTTGGTCGG | (Vigneault et al., 2004) |
|  | PADI4 | TGCATGCTGGGTCCAGATTT | GCTCAGGAACTCGTCCACAT | This study |
|  | CHD1L | GTCTCTGATGCCCTGCCTAC | GGGATGGGAACACTGCCTTTA | This study |
|  | SYCP3 | GAGAAACAAGGGAAGAAAAGG | TGCTGGAACAAAGTCAGAAAC | (Wang et al., 2012) |
|  | PADI2 | CTTCCCTCTGTCCGGTGGT | GTAGTACAGGGGTTCCGCAC | This study |
| SMAD | SMAD3 | GGAGCCGAGTACAGGAGACA | AAAGGTCCATTCAGGTGCAG; | (Zhang et al., 2015) |
| Defensin | DEFB1 | ATCCTCTAAGCTGCCGTCT | AGCATTTTACTGAGGGCGT | (Tetens et al., 2010) |
|  | DEFB3 | AAG TGA CTG CCC CTG CTT TG | GTA AAT CCT GAC CCA GCA GAC AG | This study |
|  | DEF7 | CCT GTC TGC TGG GTC AGG ATT TAC | AGG TGC CAA TCT GTC TCC TGT G | This study |
| BMP signaling | BMP4 | CCTAGCAAGAGCGCAGTCAT | CCAGATGTTCTTCGTGGTGGAA | (McLean et al., 2014) |
|  | BMP7 | TGCCACTAGCTCTTCCTGGAA | TGAGAGACCCAGGATCCAGAA | (Lee et al., 2014) |
|  | SMAD4 | AACATTGGATGGAAGGCTTCA | CCAGAGACGGGCATAGATCAC | (Zhang et al., 2015) |
|  | SMAD5 | GCAACGTTTCCTGATTCTTTCC | GGCGGGTAGGGACTATTTGG | (Lee et al., 2014) |
|  | Id1 | CTGGGATCTGGAGTTGGAGC | GGAACACACGCCGCCTCT | This study |
|  | BMPR1A | TCAGCGAACTATTGCCAAACAG | CCCATCCACACTTCTCCGTATC | (Selvaraju et al., 2013) |
| WNT signaling | WNT7a | GGT AGT CCT TCC TGC CCT TT | GTG TGT CCT GGC CTG ATT TT | (Welch-Reardon et al., 2015) |
|  | WNT10a | ATGAGTGCCAGCACCAGTTC | ATGCACTTTCTCGGAAACCTCT | This study |
|  | CTNNBL1 | AATCCGAGCTGGACCTCAATGACA | TATCATGTCCAAGCAGCCCGAGAA | (Denicol et al., 2013) |
|  | FZD7 | TTG TTT CTG GAA CCT CCT TCC GCT | GCA TCA TCC TGC AAG TCT TTG CCA | (Denicol et al., 2013) |
|  | DKK3 | GAGATGTTCCGCGAGGTTGA | ATGCACTCGTGGCTCCTTTT | This study |
|  | DKK1 | GAC TGG TGG AGG CGC TCG GA | GCT GTG CCC AGA GCC GTC AT | (Denicol et al., 2013) |
|  | DVL1 | CACTCAACATGGAGAGGCAC | GAAGTTGACGTCGTTCACCT | (Gupta et al., 2014) |
| LIF signaling | LIF | CCTCTATTACACGGCCCAGG | GTACAGCTCCACCAGCCG | This study |
|  | LIFR | AGACATGCCCTTGGAGTGTG | TCCCGCAAAAACAACCGTTC | (Kawaguchi et al., 2015) |
|  | STAT3 | CTC TCC CCA CTT CTG CCA AG | GGG GTC ACA ACT GCT GCT C | (Ilha et al., 2015) |
|  | SOCS3 | GCCACTCTCCAACATCTCTGT | TCCAGGAACTCCCGAATGG | (Vitorino Carvalho et al., 2014) |

Reference

Cagnone, G., Sirard, M.-A., 2014. The impact of exposure to serum lipids during in vitro culture on the transcriptome of bovine blastocysts. Theriogenology 81, 712-722. e713.

Denicol, A.C., Dobbs, K.B., McLean, K.M., Carambula, S.F., Loureiro, B., Hansen, P.J., 2013. Canonical WNT signaling regulates development of bovine embryos to the blastocyst stage. Sci. Rep. 3, 1266.

Gupta, P., Folger, J.K., Rajput, S.K., Lv, L., Yao, J., Ireland, J.J., Smith, G.W., 2014. Regulation and regulatory role of WNT signaling in potentiating FSH action during bovine dominant follicle selection. PloS one 9, e100201.

Ilha, G.F., Rovani, M.T., Gasperin, B.G., Antoniazzi, A.Q., Gonçalves, P.B.D., Bordignon, V., Duggavathi, R., 2015. Lack of FSH support enhances LIF–STAT3 signaling in granulosa cells of atretic follicles in cattle. Reproduction 150, 395-403.

Jiang, Z., Dong, H., Zheng, X., Marjani, S.L., Donovan, D.M., Chen, J., Tian, X.C., 2015. mRNA Levels of Imprinted Genes in Bovine In Vivo Oocytes, Embryos and Cross Species Comparisons with Humans, Mice and Pigs. Scientific reports 5.

Katz-Jaffe, M., McCallie, B., Preis, K., Filipovits, J., Gardner, D., 2009. Transcriptome analysis of in vivo and in vitro matured bovine MII oocytes. Theriogenology 71, 939-946.

Kawaguchi, T., Tsukiyama, T., Kimura, K., Matsuyama, S., Minami, N., Yamada, M., Imai, H., 2015. Generation of Naive Bovine Induced Pluripotent Stem Cells Using PiggyBac Transposition of Doxycycline-Inducible Transcription Factors. PloS one 10.

Kim, D., Park, S., Jung, Y.G., Roh, S., 2015. In vitro culture of stem-like cells derived from somatic cell nuclear transfer bovine embryos of the Korean beef cattle species, HanWoo. Reproduction, fertility, and development.

Lee, K.-B., Folger, J.K., Rajput, S.K., Smith, G.W., 2014. Temporal regulation of mRNAs for select bone morphogenetic proteins (BMP), BMP receptors and their associated SMAD proteins during bovine early embryonic development: effects of exogenous BMP2 on embryo developmental progression. Reproductive Biology and Endocrinology 12, 67.

Mamo, S., Carter, F., Lonergan, P., Leal, C.L., Al Naib, A., McGettigan, P., Mehta, J.P., Evans, A.C., Fair, T., 2011. Sequential analysis of global gene expression profiles in immature and in vitro matured bovine oocytes: potential molecular markers of oocyte maturation. BMC genomics 12, 1.

McLean, Z., Meng, F., Henderson, H., Turner, P., Oback, B., 2014. Increased MAP kinase inhibition enhances epiblast-specific gene expression in bovine blastocysts. Biology of reproduction 91, 49.

Rekawiecki, R., Rutkowska, J., Kotwica, J., 2012. Identification of optimal housekeeping genes for examination of gene expression in bovine corpus luteum. Reproductive biology 12, 362-367.

Selvaraju, S., Folger, J., Gupta, P., Ireland, J., Smith, G., 2013. Stage-specific expression and effect of bone morphogenetic protein 2 on bovine granulosa cell estradiol production: regulation by cocaine and amphetamine regulated transcript. Domestic animal endocrinology 44, 115-120.

Shimizu, T., Hirai, Y., Miyamoto, A., 2013. Expression of Cyclins and Cyclin‐Dependent Kinase Inhibitors in Granulosa Cells from Bovine Ovary. Reproduction in Domestic Animals 48, e65-e69.

Smith, C., Berg, D., Beaumont, S., Standley, N.T., Wells, D.N., Pfeffer, P.L., 2007. Simultaneous gene quantitation of multiple genes in individual bovine nuclear transfer blastocysts. Reproduction 133, 231-242.

Tetens, J., Friedrich, J.J., Hartmann, A., Schwerin, M., Kalm, E., Thaller, G., 2010. The spatial expression pattern of antimicrobial peptides across the healthy bovine udder. J Dairy Sci 93, 775-783.

Vigneault, C., McGraw, S., Massicotte, L., Sirard, M.-A., 2004. Transcription Factor Expression Patterns in Bovine In Vitro-Derived Embryos Priorto Maternal-Zygotic Transition. Biology of reproduction 70, 1701-1709.

Vitorino Carvalho, A., Eozenou, C., Healey, G.D., Forde, N., Reinaud, P., Chebrout, M., Gall, L., Rodde, N., Padilla, A.L., Delville, C.G., Leveugle, M., Richard, C., Sheldon, I.M., Lonergan, P., Jolivet, G., Sandra, O., 2014. Analysis of STAT1 expression and biological activity reveals interferon-tau-dependent STAT1-regulated SOCS genes in the bovine endometrium. Reproduction, fertility, and development.

Wang, S., Pan, Z., Zhang, Q., Xie, Z., Liu, H., Li, Q., 2012. Differential mRNA Expression and Promoter Methylation Status of SYCP3 Gene in Testes of Yaks and Cattle‐Yaks. Reproduction in domestic animals 47, 455-462.

Welch-Reardon, K.M., Wu, N., Hughes, C.C., 2015. A Role for Partial Endothelial–Mesenchymal Transitions in Angiogenesis? Arteriosclerosis, thrombosis, and vascular biology 35, 303-308.

Yang, L., Chavatte‐Palmer, P., Kubota, C., O'neill, M., Hoagland, T., Renard, J.P., Taneja, M., Yang, X., Tian, X.C., 2005. Expression of imprinted genes is aberrant in deceased newborn cloned calves and relatively normal in surviving adult clones. Mol Reprod Dev 71, 431-438.

Zhang, K., Rajput, S.K., Lee, K.-B., Wang, D., Huang, J., Folger, J.K., Knott, J.G., Zhang, J., Smith, G.W., 2015. Evidence Supporting a Role for SMAD2/3 in Bovine Early Embryonic Development: Potential Implications for Embryotropic Actions of Follistatin. Biology of reproduction, biolreprod. 115.130278.

Zhang, Y., Li, Y., Fu, C., Wang, J., Wang, H., Zan, L., 2014. Effects of bovine SMO gene polymorphisms on the body measurement and meat quality traits of Qinchuan cattle. Genetics and molecular research: GMR 13, 8105.
